# Rosiglitazone polarizes microglia and protects against pilocarpine‐induced status epilepticus

**DOI:** 10.1111/cns.13265

**Published:** 2019-11-14

**Authors:** Jing Peng, Kan Wang, Weiwei Xiang, Yan Li, Yong Hao, Yangtai Guan

**Affiliations:** ^1^ Department of Neurology Renji Hospital School of Medicine, Shanghai Jiaotong University Shanghai China; ^2^ Department of Anesthesiology Renji Hospital School of Medicine, Shanghai Jiaotong University Shanghai China

**Keywords:** epilepsy, microglia, pilocarpine, rosiglitazone, status epilepticus

## Abstract

**Aims:**

Activated microglia have been found in the forebrains and hippocampi of temporal lobe epilepsy (TLE) patients and status epileptic (SE) animal models. The peroxisome proliferator‐activated receptor γ (PPAR γ) agonist rosiglitazone has been shown to prevent microglial activation. However, its role in pilocarpine‐induced status epilepticus remains unknown. We aimed to examine the effect of the PPAR γ agonist rosiglitazone in protecting against pilocarpine‐induced status epileptic resulting from over‐activation and to explore phenotypic changes in microglia as the underlying mechanism.

**Methods:**

Male C57BL/6 mice were assigned to three groups: the control group, pilocarpine‐induced (SE) group, and rosiglitazone‐treated (SE+Rosi) group. Status epileptic mice were administered 300 mg/kg pilocarpine via intraperitoneal injection. SE+Rosi mice were administered rosiglitazone (0.1 mg/kg, i.p.) after SE. Flow cytometry, immunofluorescence staining, and quantitative real‐time PCR were used to examine the activation of and phenotypic changes in microglia in the brain and to evaluate neuroinflammation.

**Results:**

We found that the expression of proinflammatory CD86 and iNOS was increased and that the expression of antiinflammatory CD206 and Arg‐1 was decreased in the brains of pilocarpine‐induced SE mice compared to control mice. The mRNA levels of proinflammatory and antiinflammatory cytokines were not significantly changed in the brain. Rosiglitazone treatment significantly inhibited the proinflammatory polarization of microglia and rescued neuron loss in the temporal lobe and hippocampi of the brain after SE.

**Conclusion:**

Rosiglitazone reverses microglial polarization in the brains of SE mice and also affords neuroprotection against pilocarpine‐induced status epilepticus without inducing significant changes in brain inflammation.

## 1 INTRODUCTION

Epileptogenesis is the gradual development of spontaneous recurrent seizures in a normal brain.^1^ It typically occurs following various brain insults or pathological changes, including brain injury or genetic mutation, and emerging evidence suggests that microglia may play a critical role in epileptogenesis.^2^, ^3^, ^4^, ^5^, ^6^ For instance, morphologically reactive microglia have been found in the brain tissues of temporal lobe epilepsy (TLE) rodent models and human patients.^7^ As early as 8 hours following seizure, activated microglia are found in the hippocampal cornu ammonis (CA)1 and CA3 regions, the reorganization of which can cause hyper‐excitability and seizure generation.^8^, ^9^, ^10^, ^11^ Patients with hippocampal sclerosis and TLE exhibit high level of activated microglia in the hippocampi, and many of them do not respond well to antiepileptic medications.^12^, ^13^, ^14^, ^15^

Microglia are sedentary immunomodulatory cells in our central nervous system (CNS) that play critical roles in host defence and immune surveillance and harmonize innate and adaptive immune responses, which involve antigen presentation, phagocytosis, cell proliferation, cell migration, and cytokine production.^16^, ^17^ Under normal circumstances, microglial cells not only perform immune surveillance but also react to danger signals owing to distinct microglial phenotypes, including proinflammatory M1 and antiinflammatory M2 phenotypes.^18^, ^19^, ^20^ Activated microglial cells undergo morphological transformation to the M1 phenotype and then secrete proinflammatory cytokines, resulting in self‐perpetuating injury to neurons.^21^, ^22^ The other phenotype, the M2 phenotype, is neuroprotective and can promote recovery.^23^, ^24^, ^25^ Microglia‐mediated neuroinflammation has dual effects on various brain diseases, and the proinflammatory action of M1 is hypothesized to be the etiologic cause of epileptogenesis.^26^ Studies have also shown that the noninflammatory reactive‐like phenotype of microglia is adequate to drive epileptogenesis upon mTOR activation, which triggers the marked proliferation of astrocytes.^27^

Peroxisome proliferator‐activated receptor γ (PPAR γ) is a major modulator of lipid and glucose metabolism, inflammation, and organelle differentiation^28^, ^29^ and has been suggested to play important roles in many neurological disorders.^30^, ^31^ PPAR γ is a ligand‐activated transcription factor that belongs to the nuclear receptor family. Growing evidence has shown that the PPAR γ agonist rosiglitazone inhibits lipopolysaccharide (LPS)‐induced microglial activation and promotes LPS‐stimulated alterations in polarization from the deleterious M1 phenotype to the neuroprotective M2 phenotype in principal microglia.^32^ The activation of PPAR γ by pioglitazone and troglitazone reduces infarct volume by refining neurological function after middle cerebral artery occlusion in rats.^33^, ^34^ It has also been found that the PPAR γ agonist rosiglitazone imparts antidepressant‐ and anxiolytic‐like effects.^28^ Sun et al^35^ found that PPAR γ agonist prevents neuronal loss and attenuates development of spontaneous recurrent seizures (SRS) through BDNF/TrkB signaling following pilocarpine‐induced status epilepticus, and another report supports the idea that PPARγ agonist might be a potential neuroprotective agent for epilepsy by inhibiting oxidative stress and preventing astrocyte activation.^36^, ^37^ However, the role of the PPAR γ agonist rosiglitazone in protection against epilepsy from the point of microglia remains unknown. This study aimed to test whether the PPARγ agonist rosiglitazone can protect against pilocarpine‐induced status epilepticus resulting from the overactivation of microglia by reversing M1/M2 phenotypic changes.

## 2 METHODS

### 2.1 Animals

Male C57BL/6 mice aged 8‐10 weeks were purchased from Shanghai SLAC Laboratory. The animals were housed at 22°C in separate cages on a 12‐hour light‐dark cycle and provided unlimited access to food and water. All experiments were approved by the animal ethics committee of Renji Hospital and executed according to the guidelines.

### 2.2 Pilocarpine‐induced SE model

Mice were assigned to three groups, namely, the control group, pilocarpine‐induced SE group, and rosiglitazone‐treated SE group. Atropine was intraperitoneally injected at a dose of 1 mg/kg 30 minutes before pilocarpine injection to block the peripheral effects of pilocarpine. The pilocarpine‐induced group mice were administered 300 mg/kg pilocarpine (Sigma, 1538902) via intraperitoneal injection. Modeling was regarded successful when seizures reached stage 4‐5 according to Racine scale (stage 1, mouth and facial movement; stage 2, head nodding; stage 3, forelimb clonus; stage 4, rearing with forelimb clonus; and stage 5, rearing and falling with forelimb clonus).^10^

The latency and status epilepticus time were recorded. Diazepam (Sigma, D0899) was intraperitoneally injected at a dose of 5 mg/kg one hour after status epilepticus to stop seizure activity, and the mice were kept warm. The mice were then sacrificed 72 hours later. The mice with status epilepticus shorter than 60 minutes or Racine scale lower than 4 were divided into no‐SE group. The control group received saline instead of pilocarpine. The rosiglitazone‐treated group mice were administered rosiglitazone (0.1 mg/kg, i.p., Sigma, R2408) immediately after the termination of SE and at 24‐hour intervals after status epilepticus until sacrifice.

### 2.3 Flow cytometry

Flow cytometry was used to assess microglial markers. A Neural Tissue Dissociation Kit (MACS) was used to homogenize the brains of the mice according to the instructions (Miltenyi Biotec). A Percoll gradient was used to collect monocyte‐enriched cells.^38^ Cells were labeled with APC‐Cy7‐conjugated rat antimouse CD45 (BD, 557659), FITC‐conjugated rat anti‐CD11b (BD, 553310), PerCP‐Cyanine5.5‐conjugated F4/80 (Invitrogen, 45‐4801‐82), APC‐conjugated anti‐CD206 (Invitrogen, 17‐2061‐82) and BV510‐conjugated anti‐CD86 (BD, 563077) and suitable isotype controls according to the instructions (eBioscience). Before staining CD206, the cells were treated with Fixation/Permeabilization Concentrate (eBioscience, 00‐5123‐43, 00‐5223‐56) for 30 minutes and then incubated with APC‐conjugated anti‐CD206 in permeabilization buffer (eBioscience, 00‐8333‐56) for 30 minutes and analyzed on a FACSVerse cell sorter and studied with FlowJo software.

### 2.4 RT‐PCR

Quantitative real‐time polymerase chain reaction was used to measure the messenger RNA levels of specific M1 and M2 markers in animal models. Cerebral cortices and hippocampal tissues from saline‐perfused brain samples were partitioned and frozen on dry ice immediately. RNA extraction was completed using a Tissue RNA Purification Kit Plus (EZBioscience) following the manufacturer's instructions. RNA concentrations were determined using a NanoDrop spectrophotometer. First‐strand cDNA was synthesized from 500 ng RNA using HiScript III RT SuperMix for qPCR (Vazyme, R323‐01). RT‐PCR was performed using SYBR green reagents (SYBR Master Mix, Vazyme, Q411‐02) on a Light Cycler 480 II machine (Roche). A comparative Ct analysis was used, and GAPDH was used as a housekeeping reference gene. The primers for TNF‐α, IL‐1β, IL‐6, iNOS, IGF‐1, TGFβ, Ym1, IL‐10, and GAPDH are presented in Table 1.

**Table 1:** Primers sequences for quantitative real‐time polymerase chain reaction

Target genes	Forward primer sequence (5′→3′)	Reverse primer sequence (5′→3′)
TNF‐α	ATGGCCTCCCTCTCATCAGT	GTTTGCTACGACGTGGGCTA
IL‐1β	CGCAGCAGCACATCAACAAG	GTGCTCATGTCCTCATCCTG
IL‐6	ACCAGAGGAAATTTTCAATAGGC	TGATGCACTTGCAGAAAACA
iNOS	CGGACGAGACGGATAGGCAGAG	GGAAGGCAGCGGGCACATG
IGF‐1	GAGGGGCTTTTACTTCAACAAG	TACATCTCCAGTCTCCTCAGAT
TGFβ	CCAGATCCTGTCCAAACTAAGG	CTCTTTAGCATAGTAGTCCGCT
Ym1	CAGTGTTCTGGTGAAGGAAATG	ACCCAGACTTGATTACGTCAAT
IL‐10	GCTCTTACTGACTGGCATGAG	CGCAGCTCTAGGAGCATGTG
GAPDH	GACAACTTTGGCATTGTGG	ATGCAGGGATGATGTTCTG

### 2.5 Immunofluorescence staining and immunofluorescence microscopy

Mice were perfused with saline solution and formalin, and then tissues were dehydrated with 30% sucrose in PB solution at 4°C for 3‐4 days. Immunostaining was performed on free‐floating 20‐µm sections. Brain sections were blocked in 10% donkey serum for 1 hour and 1% Triton X‐100 in PBS for 20 minutes at room temperature and probed with the following primary antibodies overnight at 4°C: antimouse iNOS (BD, 610329), antirabbit iba‐1 (Wako, 019‐19741), antigoat Arg‐1 (Santa Cruz, F0915), and antimouse‐NeuN (MAB377,4739). After they were washed, the sections were treated with FITC‐labeled Alexa Fluor‐488‐ and/or Alexa Fluor‐594‐conjugated secondary antibody at a 1:1000 dilution for 1 hour. The sections were covered with DAPI Fluoromount‐G® (Southern Biotech). The stained cells were visualized and photographed by a confocal microscope.

### 2.6 Statistical methods

All results are presented as the mean ± SEM. Student's *t *test or Mann‐Whitney *U* (nonparametric tests) was used for two‐group comparisons. For multiple groups, one‐way ANOVA or Kruskal‐Wallis test (nonparametric tests) followed by the Bonferroni test was used. Nonparametric tests were conducted because of the nonnormal distribution and nonhomogeneity of variance. The results were deemed statistically significant at *P* ≤ .05. Statistical analyses were performed using SPSS software (v. 24.0). All figures were made by GraphPad Prism software (v. 7.0).

## 3 RESULTS

### 3.1 Rosiglitazone rescued neurons loss in SE mouse brains

**Figure 1:**
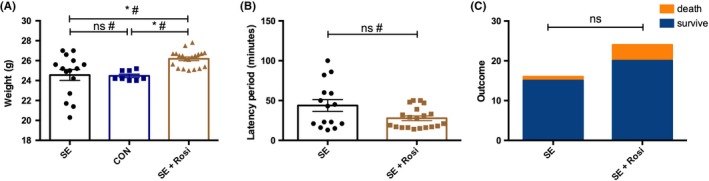
Data from the mice assessed in our study. (A) The weights of the mice in the SE (n = 15), control (n = 8), and SE+Rosi groups (n = 20) were compared, and the weights of the mice in the SE+Rosi group were higher than those of the mice in the other groups. (B) The latencies to SE development in the SE and SE+Rosi groups were similar. (C) After SE, the outcomes of the mice in the rosiglitazone treatment group were not better than those of the mice in the SE group. *P < .05; ns indicates not significant; # indicates a nonparametric test

We administered pilocarpine (300 mg/kg, once, i.p.) to mice to induce status epilepticus. In this model, 24.7% of the mice developed SE. The survival rate was 93.3% in the SE group. In another group of mice to which we administered rosiglitazone (0.1 mg/kg, i.p., once every 24 hours for 72 hours), the survival rate was 83.3%, which was not significantly different from that of SE mice without rosiglitazone treatment (Figure 1C). We also found that the weight of the SE+Rosi mice was higher than that of the SE and control mice (Figure 1A). The latency of SE development was comparable between the SE and SE+Rosi mice (Figure 1B).

**Figure 2:**
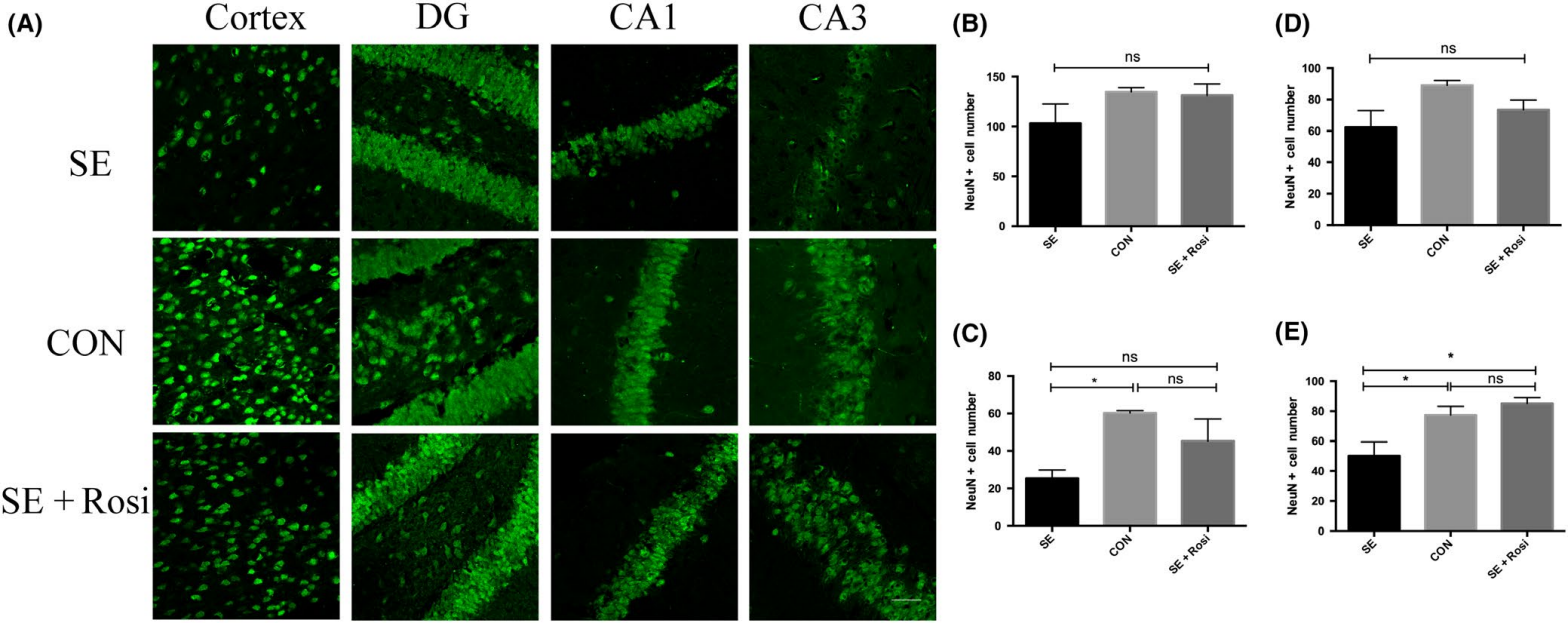
Neuron marker‐NeunN staining in the temporal lobe cortex and hippocampal tissues 3 d after SE or SE plus rosiglitazone administration. (A) Immunofluorescence staining for neuron (green) in the SE (n = 4), control (n = 3), and SE+Rosi (n = 3) groups. The number of neurons in temporal lobe cortex and dentate gyrus (DG) region, cornu ammonis (CA)1, and CA3 hippocampi in SE group was lower than control group and SE+Rosi group. (B‐E) The number of NeuN+ neurons was statistically analyzed. There was no significant difference in temporal lobe cortex among three groups (B). The NeuN+ pyramidal neurons in DG region were statistically decreased in SE group than control group and were higher in SE+Rosi group (C). The NeuN+ neurons of CA1 hippocampi were decreased in SE group, but not in SE+Rosi group without statistical difference (D). The NeuN+ neurons of CA3 hippocampi were significantly decreased in SE group than control group and SE+Rosi group (E). *P < .05; ns indicates not significant; Scale bar = 50 μm

In order to observe the protective effect of rosiglitazone on SE brains, we stained the neurons by NeuN immunofluorescence staining and found that the NeuN+ cells in the temporal lobe cortex and hippocampal tissues 3 days after SE were decreased than control mice, especially in dentate gyrus (DG) region and CA3 hippocampi (Figure 2A‐E). After rosiglitazone administration, the number of NeuN+ cells was increased 3 days after SE especially in CA3 hippocampi (Figure 2E). Although the rising of NeuN+ neurons number after rosiglitazone treatment was not so significantly in temporal lobe cortex, DG regions, and CA1 hippocampi (Figure 2B‐D), these results could reflect the protective effect of rosiglitazone on SE brains. In summary, we speculate that rosiglitazone may play key roles in neuron protection by reversing microglial polarization.

### 3.2 Pilocarpine‐induced status epilepticus resulted in increased CD86 expression but decreased CD206 expression in microglia in the forebrain, and rosiglitazone reversed these changes

**Figure 3:**
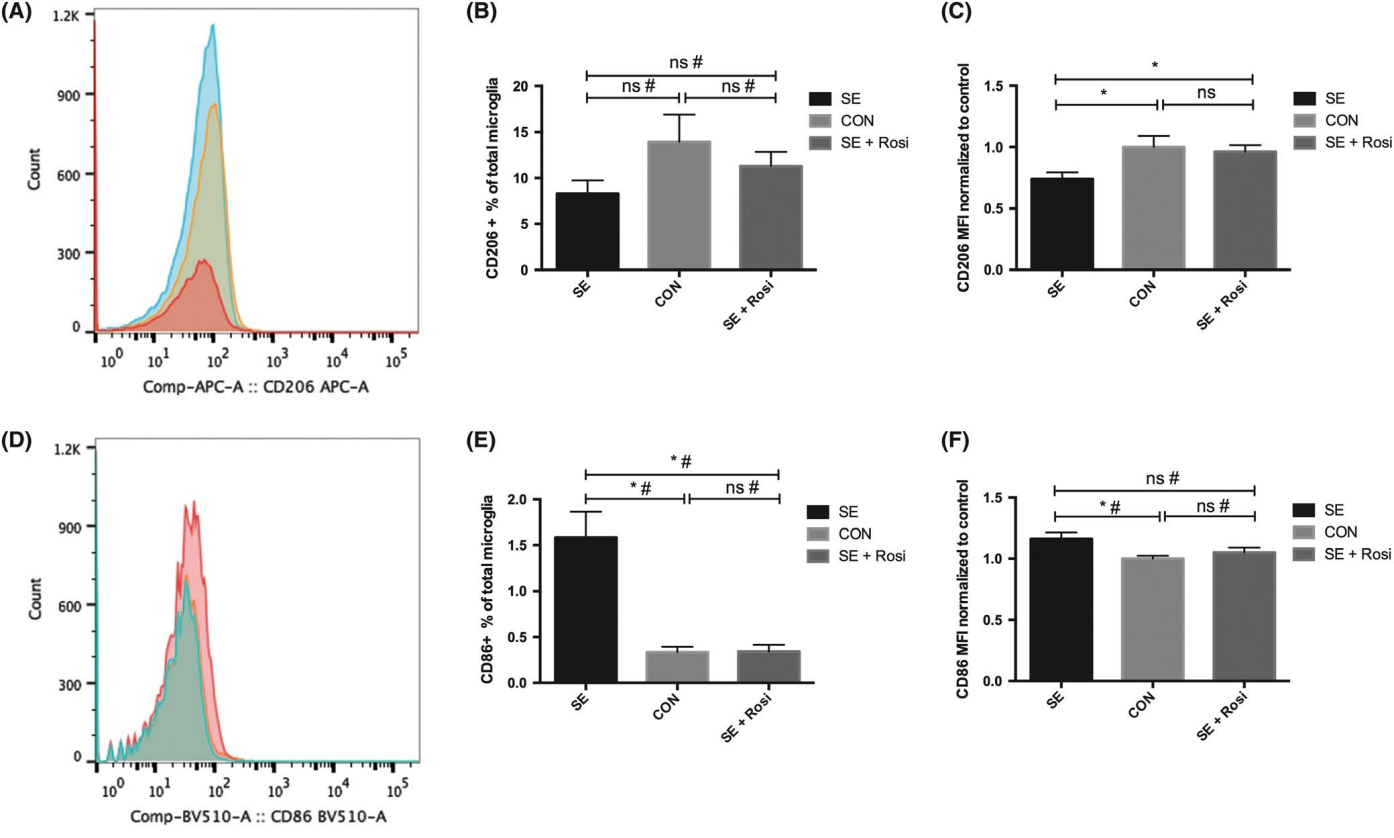
Microglial‐specific expression of the M1 phenotype marker CD 86 and the M2 phenotype marker CD 206 was detected by flow cytometry in the forebrains of mice 3 d after SE induced by pilocarpine or SE plus rosiglitazone administration. (A, D) Histogram curves of the expression of the M2 phenotype marker CD206 and the M1 phenotype marker CD86 in microglial cells comparing control (blue line, n = 8), SE (red line, n = 8), and SE+Rosi (yellow line, n = 8) mice, as determined by flow cytometry. In addition, CD 86 expression was increased significantly in the SE group compared to the control group, and CD206 expression was decreased significantly in the SE group compared to the control group. Rosiglitazone reversed these changes. (B, E) The percentage of CD206+ or CD86+ microglia relative to total microglia was calculated, and the same trend as that in A and D was observed. (C, F) The median fluorescence intensity (MFI) of CD206 or CD86 normalized to that of the control was also statistically analyzed, and the trend was similar, although some data did not reach statistical significance. *P < .05; ns indicates not significant; # indicates a nonparametric test

To further examine the phenotypic profiles of microglia, flow cytometry was used to separate microglia (CD45‐intermediate) from other leukocytes (CD45‐high) isolated from forebrain homogenates. The expression of CD86 and CD206 was examined, and it was found that there were distinctive changes in the brains of pilocarpine‐induced SE mice (Figure 3A, D). The median fluorescence intensity (MFI) of CD206 was decreased significantly in the brains of SE mice compared to control mice but not in SE+Rosi mice (Figure 3A‐C). Meanwhile, the expression of CD86 was examined, and we found that the MFI of CD86 and the percentage of CD86+ microglia were significantly increased in the brains of SE mice but not in the brains of SE+Rosi mice (Figure 3D‐F). These results suggest that proinflammatory microglia are activated, while the number of antiinflammatory microglia is decreased in the brains of SE mice and that rosiglitazone treatment reverses these phenotypic changes in microglia in pilocarpine‐induced status epilepticus.

### 3.3 Rosiglitazone treatment did not significantly change the mRNA expression of proinflammatory cytokines in the forebrains and hippocampi of SE mice

**Figure 4:**
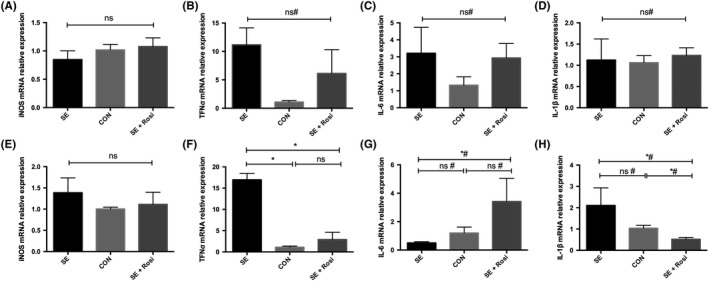
M1 phenotype‐associated cytokines were detected using quantitative real‐time PCR in temporal lobe and hippocampal tissues 3 d after SE or SE plus rosiglitazone administration. (A‐D) The mRNA levels of the M1 phenotype‐associated cytokines iNOS, TNF‐α, IL‐6, and IL‐1β in temporal lobe tissues; no significant difference was observed. (E‐H) M1 phenotype‐associated cytokines in hippocampal tissues were measured, and rosiglitazone inhibited the elevation of TNF‐α and IL‐1β, but not iNOS or IL‐6, in hippocampal tissues. *P < .05; ns indicates not significant; # indicates a nonparametric test. SE group (n = 4), control group (n = 5), and SE+Rosi group (n = 5)

Next, we examined the mRNA expression of proinflammatory cytokines in the brains of pilocarpine‐induced status epilepticus mice. We found no significant differences in the expression of iNOS, TNF‐α, IL‐6, or IL‐1β in temporal lobe tissues among the three different groups (see Figure 4A‐D). However, the TNF‐α of SE group is significantly higher than no‐SE group in temporal lobe tissues (Figure S1B). While the mRNA expression of the inflammatory cytokine TNF‐α was significantly increased in the hippocampi of SE mice compared to control mice and no‐SE (Figure S1F), and this change was reversed in SE+Rosi mice (Figure 4F). However, the mRNA expression of iNOS, IL‐6, and IL‐1β was not significantly changed in the hippocampi across all groups (Figure 4E,G,H).

### 3.4 Rosiglitazone suppressed the increased mRNA expression of antiinflammatory cytokines in the forebrains and hippocampi of SE mice

**Figure 5:**
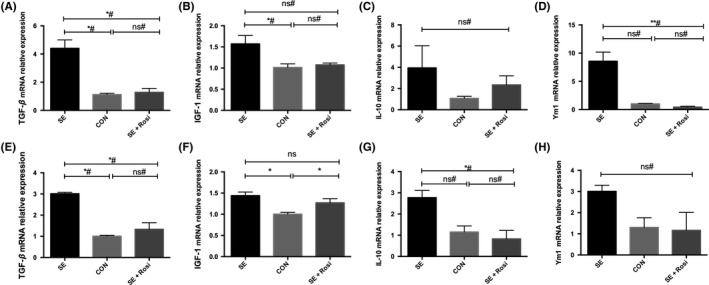
M2 phenotype microglia‐associated cytokines were detected using quantitative real‐time PCR in temporal lobe and hippocampal tissues 3 d after pilocarpine or rosiglitazone administration. (A‐D) The mRNA levels of the M2 phenotype‐associated cytokines TGF‐β, IGF‐1, IL‐10, and Ym1 in temporal lobe tissues. The mRNA levels of Ym1, TGF‐β, and IGF‐1, but not IL‐10, were increased in the SE group, and rosiglitazone reversed these changes. (E‐H) M2 phenotype‐associated cytokines in hippocampal tissues were also measured, and rosiglitazone inhibited the elevation of TGF‐β and IL‐10, but not IGF‐1 or Ym1, in hippocampal tissues. *P < .05; **P < .01; ns indicates not significant; # indicates a nonparametric test. SE group (n = 4), control group (n = 5), and SE+Rosi group (n = 5)

We also quantified the mRNA expression of antiinflammatory cytokines. Unexpectedly, we found that the mRNA expression of TGF‐β and IGF‐1 was increased in temporal lobe and hippocampal tissues of SE mice compared to control mice but that rosiglitazone suppressed these changes in TGF‐β (Figure 5A,E), but not IGF‐1 despite similar trend (Figure 5B,F). The expression of IL‐10 and Ym1 in temporal lobe and hippocampal tissues of SE mice was not significantly changed, but higher than no‐SE mice in hippocampal tissues (Figure S2G), while IL‐10 and Ym1 mRNA expression has an increasing trend in the temporal lobes and hippocampal tissues of SE mice which was suppressed by rosiglitazone‐treatment (Figure 5C,D,G,H).

### 3.5 SE induced an increase in iNOS expression in microglia but not in rosiglitazone‐treated SE brains

**Figure 6:**
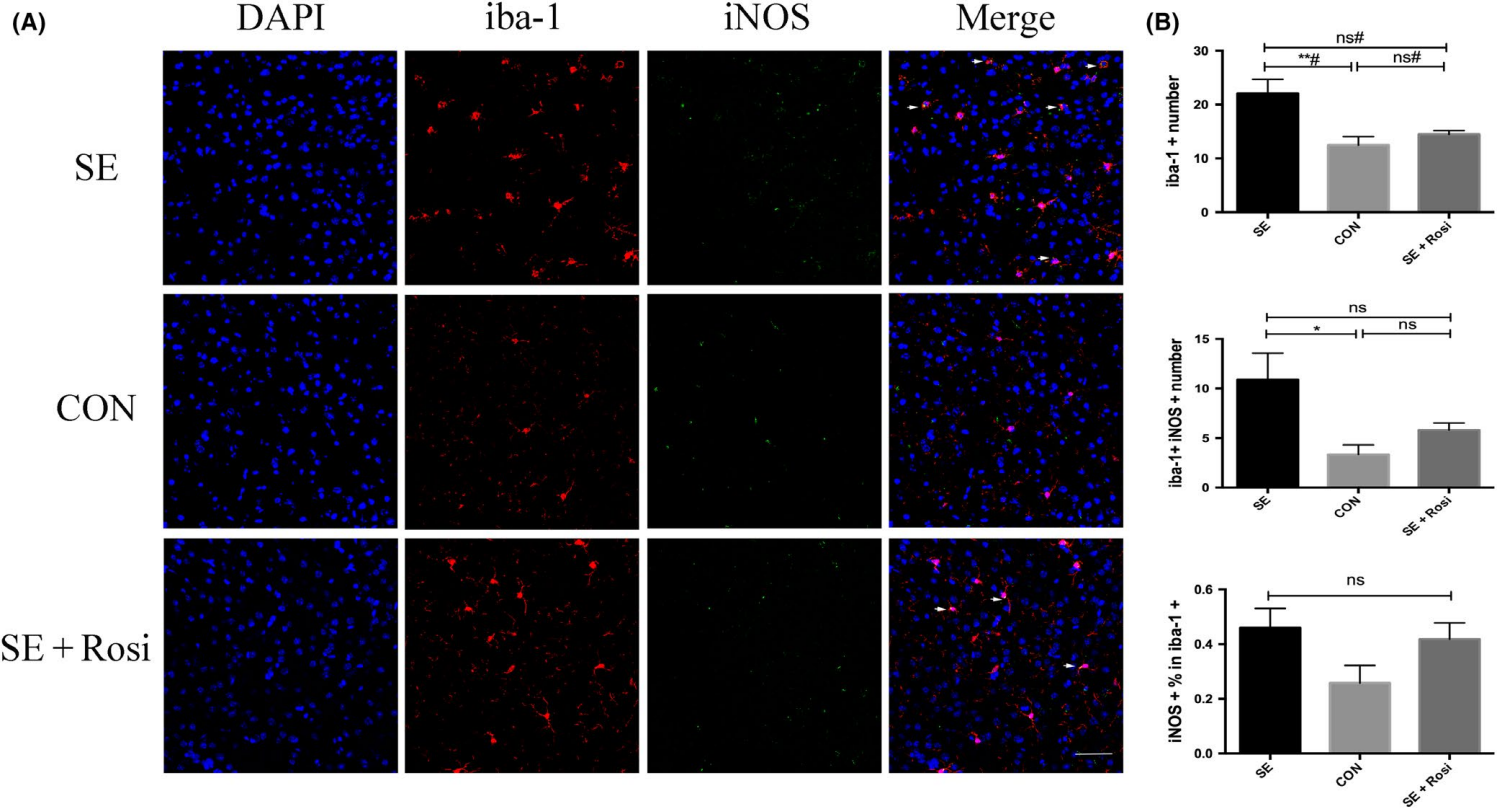
An M1 phenotype marker was detected using immunofluorescence staining in the temporal lobe 3 d after pilocarpine or rosiglitazone administration. (A) Immunofluorescence staining for DAPI (blue), iba‐1 (red), and iNOS (green) in the SE (n = 8), control (n = 5), and SE+Rosi (n = 7) groups. The number of iba‐1+ (red) microglia was increased in the SE group, and this increase was inhibited in the SE+Rosi group. In addition, the numbers of iba1+ and iNOS+ (green) microglia were also elevated in the SE group compared to the control group, and these changes were reversed after rosiglitazone administration for 3 d. (B) The immunofluorescence staining results were statistically analyzed. Upper panel: the number of iba‐1+ microglia in SE mice was higher than that in control mice and was decreased after rosiglitazone administration, although the difference was not significant. Middle panel: the number of iba‐1+ and iNOS+ microglia in SE mice was higher than those in control mice, and this change was reversed by treatment with rosiglitazone. Lower panel: there were no significant differences in the percentages of iNOS+ microglia relative to iba‐1+ microglia among the three groups. *P < .05; **P < .01; ns indicates not significant; # indicates a nonparametric test. Scale bar = 50 μm

Next, we further examined proinflammatory iNOS expression in microglia using immunofluorescence staining in SE mice. We found that microglia was significantly activated with the number of iNOS+ Iba‐1+ microglia increasing in the brains of SE mice compared to control mice and no‐SE mice (Figure S3B, C); however, rosiglitazone significantly reduced the activation of microglia and the expression of iNOS in microglia (Figure 6A,B). Additionally, the percentage of iNOS+ Iba‐1+ microglia was not significantly changed in SE mouse brains or rosiglitazone‐treated SE mouse brains (Figure 6A,B). These data suggest that rosiglitazone can suppress proinflammatory iNOS expression in microglia, which is increased in SE mouse brains.

### 3.6 Rosiglitazone increased Arg‐1 expression in microglia in SE mouse brains

**Figure 7:**
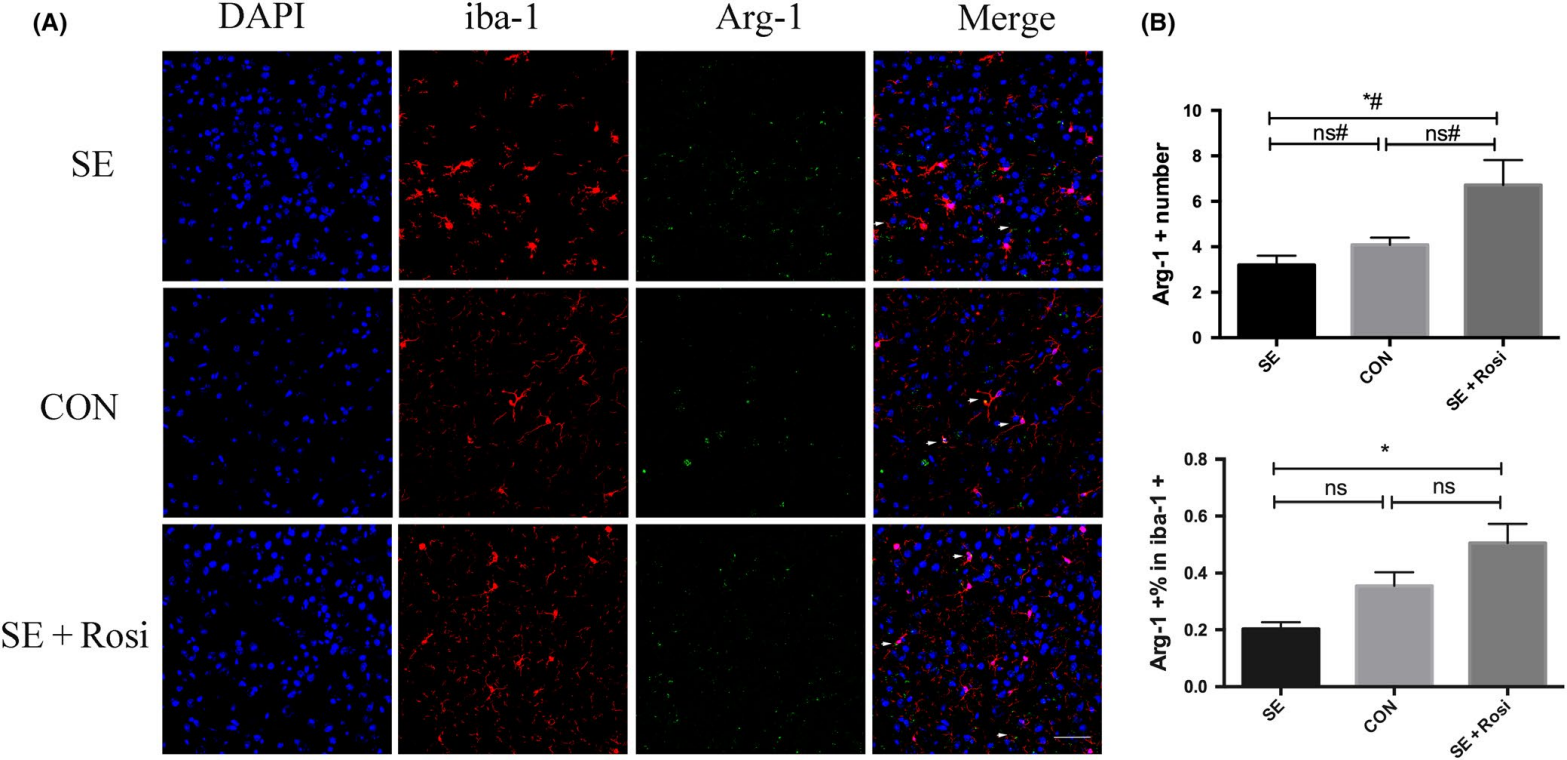
An M2 phenotype microglia marker was detected using immunofluorescence staining in the temporal lobe 3 d after pilocarpine or rosiglitazone administration. (A) Immunofluorescence staining for DAPI (blue), iba‐1 (red), and Arg‐1 (green) in the SE (n = 8), control (n = 5), and SE+Rosi (n = 7) groups. The number of iba‐1+ (red) microglia was increased in the SE group and was decreased in the SE+Rosi group. In addition, the number of iba1+ and Arg‐1+ (green) microglia was decreased in the SE group compared to that in the control group and was increased after rosiglitazone administration for 3 d. (B) The immunofluorescence staining results were statistically analyzed. Upper panel: the number of iba‐1+ and Arg‐1+ microglia in SE mice was lower than that in control mice, and this change was reversed by treatment with rosiglitazone. Lower panel: the percentage of Arg‐1+ microglia relative to iba‐1+ microglia in the SE group was lower than that in the control group, but this change was reversed by treatment with rosiglitazone. * *P* < .05; ns indicates not significant; # indicates a nonparametric test. Scale bar = 50 μm

We also tested the expression of antiinflammatory Arg‐1 (Arginase‐1) in microglia of SE mice. We found that SE did not significantly change the expression of Arg‐1 in microglia; however, rosiglitazone significantly increased the number and percentage of Arg‐1+ microglia in rosiglitazone‐treated SE mice compared to SE mice (Figure 7A,B). These data suggest that rosiglitazone can upregulate antiinflammatory Arg‐1 expression in microglia in SE mouse brains.

Taken together, our results suggest that SE induces the polarization of proinflammatory microglia but reduces the polarization of antiinflammatory microglia in the brain. Rosiglitazone reverses microglial polarization in the brains of SE mice and affords neuroprotection against pilocarpine‐induced status epilepticus without significantly altering inflammation in the brain.

## 4 DISCUSSION

Microglia are known to play a critical role in maintaining brain homeostasis.^39^ The polarization of microglia has been suggested to play pivotal roles in several different neurological disorders.^40^, ^41^, ^42^, ^43^, ^44^ The activation of microglia after seizure in the epileptic brain has been investigated in several studies.^37^, ^45^, ^46^ Furthermore, the role of activated microglia in epileptogenesis has been determined in both inflammatory and noninflammatory processes.^27^ Consistent with the previous studies, we found that M1 microglia in the forebrain increased significantly after acute pilocarpine‐induced seizure (3 days after SE), as determined by flow cytometry and immunofluorescence. More importantly, our study showed that proinflammatory CD86 was significantly elevated; flow cytometry and immunofluorescence identified significantly increased proinflammatory iNOS expression in microglia after acute pilocarpine‐induced seizure. In addition, we used quantitative real‐time PCR to detect cytokine changes in temporal lobe cortical and hippocampal tissues; we found that although TNF‐α and IL‐6 mRNA levels were increased in temporal lobe tissues, the difference did not reach statistical significance and that the mRNA expression of iNOS and IL‐1β was not changed 3 days after SE seizures.

On the other hand, we observed another antiinflammatory phenotypic change 3 days after pilocarpine‐induced SE using flow cytometry and immunofluorescence staining. To our surprise, the mRNA levels of antiinflammatory microglia‐associated cytokines were elevated in the forebrains of SE mice; this was not consistent with the flow cytometry results. In temporal lobe and hippocampal tissues from SE mice, TGF‐β and IGF‐1 mRNA levels were significantly higher than those in control mice. The difference in IL‐10 and Ym1 expression between SE mice and control mice did not reach statistical significance. Taken together, our results suggested that SE induced the polarization of proinflammatory microglia but reduced the polarization of antiinflammatory microglia in the brain. Rosiglitazone reversed microglial polarization in the brains of SE mice and afforded neuroprotection against pilocarpine‐induced status epilepticus without significantly altering inflammation in the brain. The discrepancy in the RT‐PCR results of mRNA expression in the brain may have been due to the various cell types, including neurons, astrocytes, microglia, and endothelial cells, in the brain tissue samples. Recently, it has been shown that activated microglia play a primary role in the production of cytokines. The expression of proinflammatory cytokines increased 3 days, but not 21 days, after pilocarpine‐induced SE, however, the expression of antiinflammatory cytokines was also increased in the epileptic brain,^47^ indicating a complex microglial inflammatory response during epileptogenesis.

Several researches showed that the activation of microglia is most significant on 3 days after status epilepticus induced by either kainic acid or pilocarpine; Microglial‐specific expression of M1 and M2 markers significantly increases at the acute (3 days post‐SE) time point in pilocarpine‐induced SE forebrains. Therefore, we chose 3 days post‐SE as the time point to observe the effect of rosiglitazone on microglia polarization and SE brain damage. Furthermore, there are several drawbacks in our study: we have not observed the dynamic changes in microglia polarization at various time points after SE and explored the further specific underlying mechanisms of rosiglitazone's effect on microglia polarization after SE which needs to be explored in the future.

There is growing evidence that the PPARγ agonist rosiglitazone prevents microglial activation, promotes microglial antiinflammatory polarization, and suppresses inflammatory cytokines in inflammation‐related diseases, such as MS, EAE, and Parkinson's disease.^32^ However, the protective effect of rosiglitazone after SE needs to be clarified. Our study revealed that the PPARγ agonist rosiglitazone can suppress the activation of microglia by flow cytometry and immunofluorescence staining for Iba‐1. To our surprise, rosiglitazone also inhibited proinflammatory microglial polarization, during which the expression of CD206 and Arg‐1 is increased.^32^ In addition, we observed the loss of neurons in temporal lobe and hippocampal tissues after SE, consistent with the previous study.^48^ After rosiglitazone administration, the number of NeuN+ cells was increased 3 days after SE, especially in CA3 hippocampi. These results all indicate that rosiglitazone plays an important role in neuroprotection after acute seizure. We infer that the shift of microglia phenotype from M2 to M1 could be one of the key mechanisms of rosiglitazone to protect from SE brain damage. On the other hand, there may be other mechanisms for PPARγ to be involved in epileptic brain injury, especially concerning microglia, which needs to be explored further in the future.

In conclusion, rosiglitazone can regulate the polarization of microglia in SE mice and protect against pilocarpine‐induced status epilepticus without significantly altering inflammation in the brain.

## CONFLICT OF INTEREST

The authors declare no conflict of interest.

## ETHICAL APPROVAL

This study was approved by the ethics committee of Renji Hospital, Shanghai Jiao Tong University School of Medicine.

## CONSENT FOR PUBLICATION

Available upon request.

## Data Availability

Not applicable.
