# Supplementary figures and images for: Rosiglitazone polarizes microglia and protects against pilocarpine‐induced status epilepticus
Source: CNS Neurosci Ther. 2019 Nov 14;25(12):1363–72. doi: 10.1111/cns.13265 (PMC6887926; doi:10.1111/cns.13265)

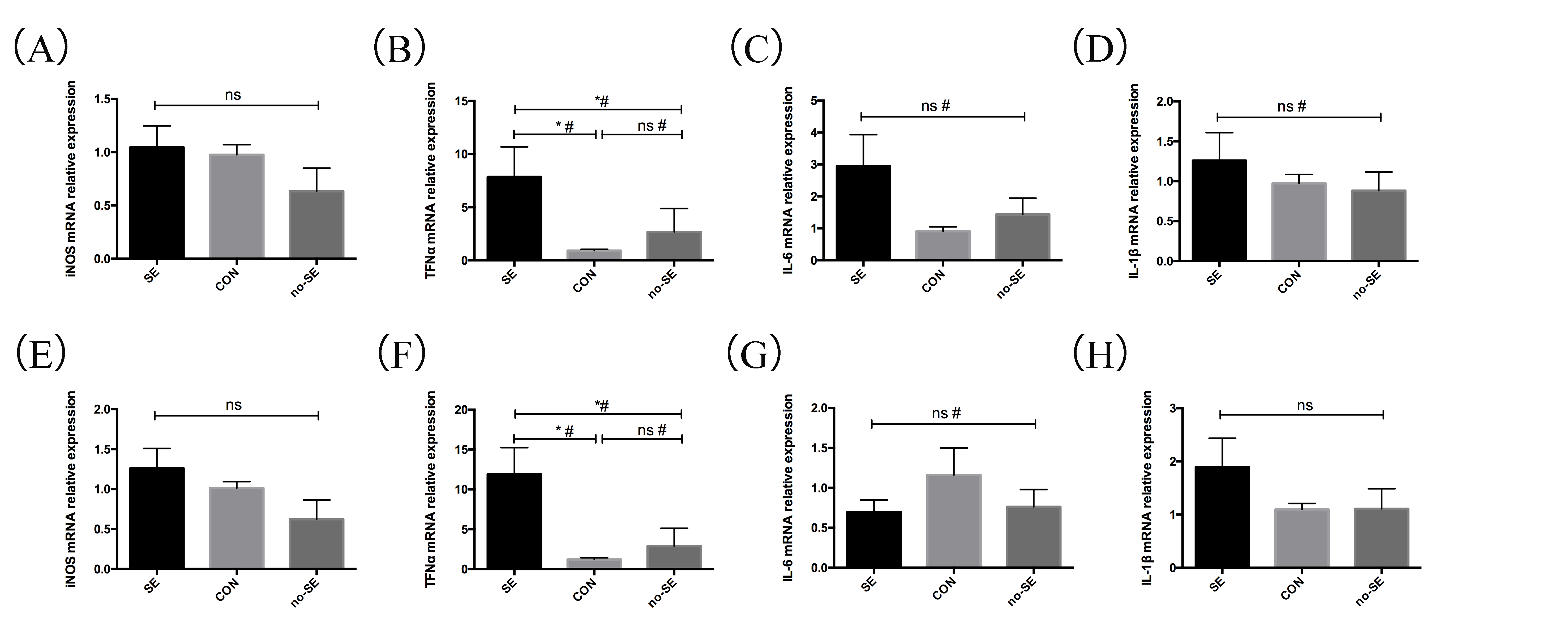

Supplement: Supplementary file 1 [file CNS-25-1363-s001.tif]

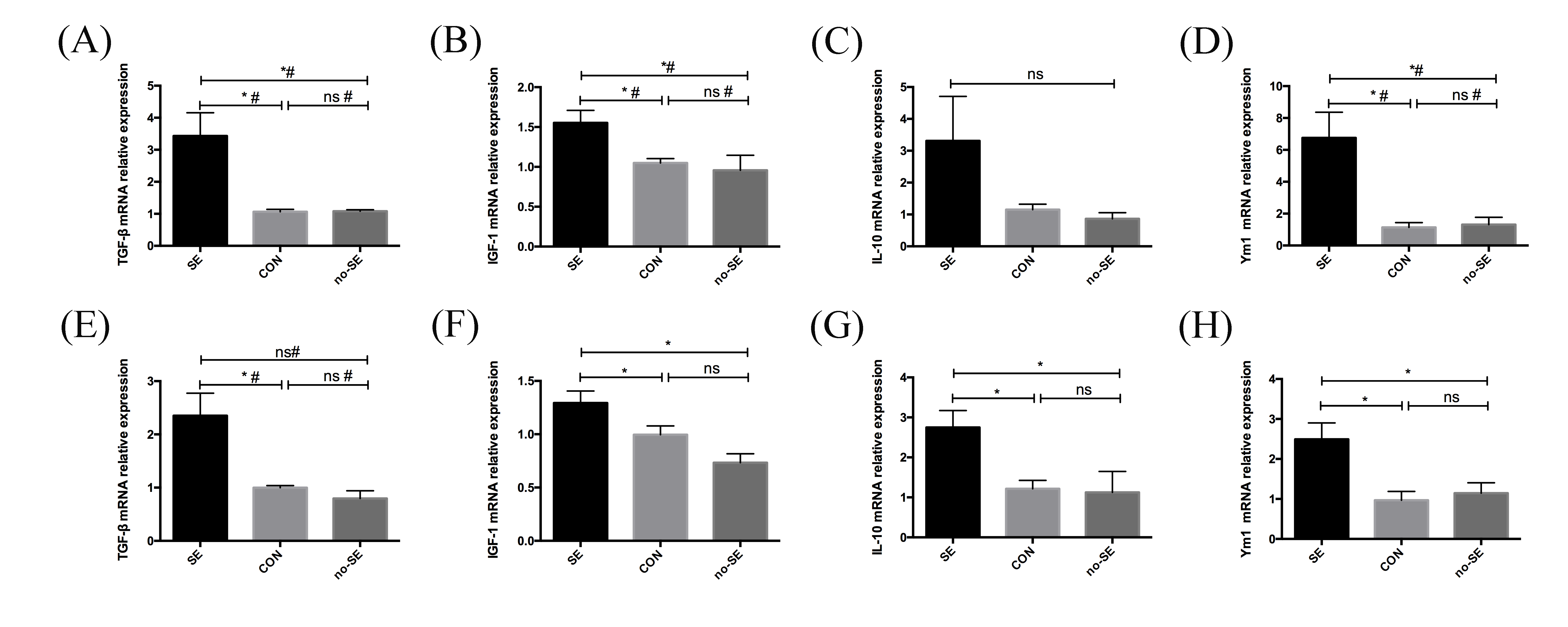

Supplement: Supplementary file 2 [file CNS-25-1363-s002.tif]

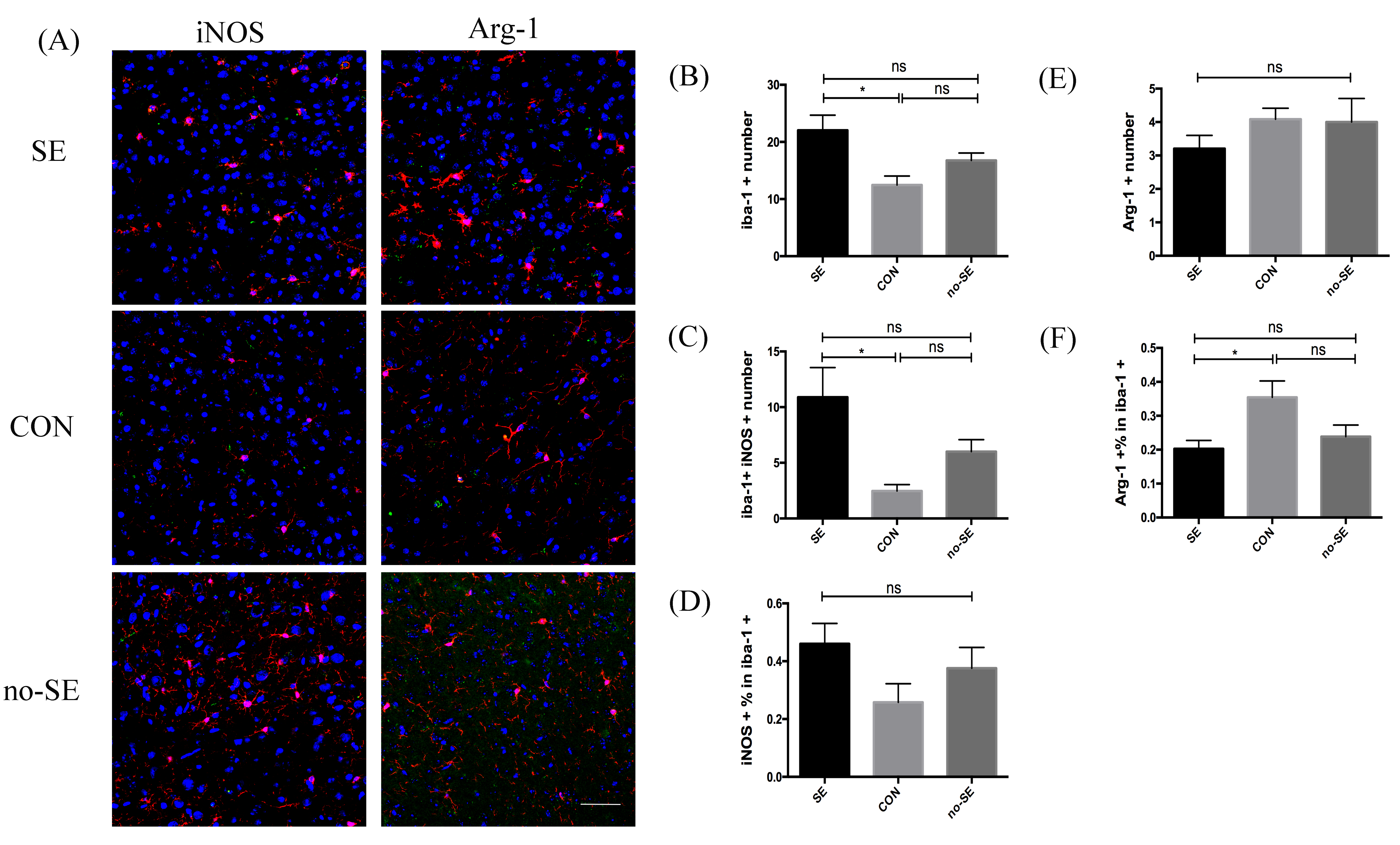

Supplement: Supplementary file 3 [file CNS-25-1363-s003.tif]
