# Supplementary material for: Rosiglitazone polarizes microglia and protects against pilocarpine‐induced status epilepticus
Source: CNS Neurosci Ther. 2019 Nov 14;25(12):1363–72. doi: 10.1111/cns.13265 (PMC6887926; doi:10.1111/cns.13265)
Supplement: Supplementary file 4 [file CNS-25-1363-s004.docx]

**Supplementary figure 1**

M1 phenotype-associated cytokines were detected using quantitative real-time PCR in temporal lobe and hippocampal tissues 3 days after SE. (A-D) The mRNA levels of the M1 phenotype-associated cytokines iNOS, TNF-α, IL-6 and IL-1β in temporal lobe tissues; TNF-α levels in SE group were significantly higher than control group and no-SE group, no significant difference was observed about other M1 cytokines, but with a higher tendency in SE group. (E-F) M1 phenotype-associated cytokines in hippocampal tissues were measured. TNF-α levels in SE group were significantly higher than control group and no-SE group, other M1 cytokines including iNOS, IL-6 and IL-1β were not significantly changed with a higher elevation in hippocampal tissues. *P<0.05; ns indicates not significant; # indicates a non-parametric test. SE group (n=6), control group (n=6), no-SE group indicates mice with status epileptic less than 1 hour (n=2) or without status epilepticus (n=2).

**Supplementary figure 2**

M2 phenotype microglia-associated cytokines were detected using quantitative real-time PCR in temporal lobe and hippocampal tissues 3 days after SE. (A-D) The mRNA levels of the M2 phenotype-associated cytokines TGF-β, IGF-1, IL-10 and Ym1 in temporal lobe tissues. The mRNA levels of TGF-β, IGF-1 and Ym1, were increased in the SE group than control group and no-SE group. IL-10 levels were not significantly higher in SE group. (E-F) M2 phenotype-associated cytokines in hippocampal tissues were also measured, TGF-β, IL-10, IGF-1 and Ym1 were all higher in SE group than control group and IL-10, IGF-1 and Ym1 were all lower in no-SE group than SE group. * P<0.05; ns indicates not significant; # indicates a non-parametric test. SE group (n=6), control group (n=6), no-SE group (n=4).

**Supplementary figure 3**

M1 and M2 phenotype microglia marker was detected using immunofluorescence staining in the temporal lobe 3 days after SE . (A) Immunofluorescence staining for DAPI (blue), iba-1 (red), iNOS(left, green) and Arg-1 (right, green) in the SE (n=8), control (n=5) and no-SE (n=4) groups. The number of iba-1+ (red) microglia was increased in the SE group, but not in no-SE group (B). The number of iba1+(red) and iNOS+ (green) microglia was increased in the SE group, but not in no-SE group(C). The percentage of iNOS+ microglia relative to iba-1+ microglia was not significantly changed among three groups, but with a higher tendency in SE group(D). The number of iba-1+(red) and Arg-1+(green) microglia in SE mice was lower than that in control mice, but without significant change(E). The percentage of Arg-1+ microglia relative to iba-1+ microglia in the SE group was significantly lower than that in the control group, but not in no-SE group. (F) * P<0.05; ns indicates not significant. Scale bar = 50 μm.
